# Sirtuin 3 is required for the dexmedetomidine‐mediated alleviation of inflammation and oxidative stress in nephritis

**DOI:** 10.1002/iid3.1135

**Published:** 2024-01-10

**Authors:** Kai Lu, Xinlong Li, Jie Wu

**Affiliations:** ^1^ Department of Anesthesiology, Sir Run Run Shaw Hospital, School of Medicine Zhejiang University Hangzhou China

**Keywords:** apoptosis, dexmedetomidine, inflammation, nephritis, oxidation, renoprotection, sirtuin 3

## Abstract

**Introduction:**

Although sirtuin 3 (SIRT3) is known to be involved in dexmedetomidine (DEX)‐mediated alleviation of renal ischemia and reperfusion injury, the influence of the association between DEX and SIRT3 on nephritis development remains unclear. In this study, the role of SIRT3 in DEX‐mediated amelioration of inflammation and oxidative stress in nephritis as well as the possible underlying mechanism were explored in vivo and in vitro.

**Methods:**

An animal model of glomerulonephritis was generated by injecting mice with interferon‐alpha (IFNα)‐expressing adenoviruses, and periodic acid–Schiff staining was then used to reveal pathogenicity‐related changes in the renal tissue. Additionally, human embryonic kidney cells (HEK293) and renal mesangial cells (RMCs) were treated with IFNα to establish cell models of inflammation in vitro.

**Results:**

DEX administration alleviated glomerulonephritis in the animal model and upregulated SIRT3 expression in the renal tissue. *SIRT3* knockdown inhibited the renoprotective effects of DEX against nephritis. IFNα induced inflammation, oxidative stress, and apoptosis in the RMCs and HEK293 cells and reduced their growth, as evidenced by the evaluation of cytokine levels (enzyme‐linked immunosorbent assay), reactive oxygen species generation, catalase and superoxide dismutase activities, nuclear factor‐erythroid factor 2‐related factor 2/heme oxygenase‐1 signal transduction, apoptotic cell proportion, and cell viability. In addition to promoting SIRT3 expression, DEX inhibited IFNα‐induced inflammation, oxidative stress, and apoptosis in these cells and promoted their viability. *SIRT3* knockdown partially reversed the beneficial effects of DEX on RMCs and HEK293 cells.

**Conclusions:**

Our results suggest that DEX exhibits renoprotective activity during nephritis progression, protecting renal cells against inflammatory injury by promoting SIRT3 expression.

## Graphical abstract

Dexmedetomidine (DEX) prevents nephritis by upregulating although sirtuin 3 (SIRT3) expression. The results of this study offer supporting information on the potential mechanism of DEX in its protection of the kidneys in lupus nephritis as well as the involvement of SIRT3 in this process.

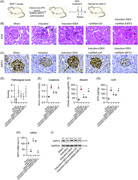

## 1 INTRODUCTION

Systemic lupus erythematosus (SLE), an immune system‐associated disorder, is characterized by the heterogeneity of its extensive clinical presentations.^1^ One of the most severe complications of SLE is lupus nephritis (LN), which is a leading prognostic predictor of poor outcomes in afflicted patients.^2^, ^3^ The most common symptoms of LN are tubulointerstitial inflammation and glomerular phlegmonosis, which are likely caused by the deposition of immune complexes in the kidneys.^4^, ^5^ Subsequently, type I interferon (IFNα) is produced, and a series of inflammatory reactions are activated, which are critical for the course of the illness.^6^, ^7^ However, the length of time needed for numerous varieties of lupus‐susceptible mice to develop LN spontaneously hampers their use as animal models. Additionally, the onset of the disease is unstable, which greatly impedes studies on its outcomes and the development of effective therapeutic interventions. By contrast, the delivery of exogenous IFNα to lupus‐susceptible mice promotes rapid manifestation of the clinical features of LN. Moreover, the onset of the disease can be controlled to occur at the same time in many animals, allowing for the reproducible observation of its steady progress. Therefore, IFNα‐accelerated LN development is a useful model for studying the pathogenic mechanisms of the disease and testing new therapeutic strategies.^8^

Immunocytes, particularly plasmacytoid dendritic cells, are activated by immune complexes deposited in the kidneys, influencing the activation of genes that lie downstream of the IFNα pathway.^9^ Renal mesangial cells (RMCs) are resident mesenchymal stem cells that participate in the progression of LN.^10^, ^11^ It is known that the IFNα molecules produced by resident renal cells, including RMCs, can worsen autoimmune kidney damage.^12^, ^13^ However, the mechanism underlying the regulation of IFNα signaling in RMCs has still not been completely elucidated.

The seven mammalian sirtuin subtypes (SIRT1–SIRT7) share the same conserved catalytic core region consisting of 275 amino acids but differ in their subcellular localization and physiological functions.^14^ Among them, SIRT1 is widely documented to exert renoprotective effects, inhibiting inflammation, cell apoptosis, and fibrosis in the kidneys. Therefore, SIRT1 activation has the potential to be a novel therapeutic target in the treatment of patients with chronic kidney diseases, such as diabetic nephropathy.^15^ As a nicotinamide adenine dinucleotide (NAD^+^)‐dependent histone deacetylase, SIRT3 plays regulatory roles in several mammalian physiological processes, such as metabolism and aging.^16^, ^17^ Deacetylated mitochondrial matrix proteins, such as cyclophilin D and superoxide dismutase 2 (SOD2), have protective effects on the mitochondria, thus completing the process of SIRT3 activation.^18^, ^19^ SIRT3 activation or overexpression has also been shown to have a protective effect on myocardial cells by preventing the damage caused by H_2_O_2_. Additionally, both the deacetylation of cyclophilin D and inhibition of the opening of mitochondrial permeability transition pores have been shown to alleviate myocardial ischemia–reperfusion (I/R) injury.^18^ In the kidneys, SIRT3 also acts as a stimulant of mitochondrial activity, thereby inhibiting renal oxidative stress in animals fed fatty foods.^19^ It has been shown in models of acute kidney injury that the mitochondrial dynamics are better recovered when SIRT3 is overexpressed.^20^ In another study, SIRT3 expression was shown to be upregulated by dexmedetomidine (DEX), and the treatment attenuated renal I/R injury by inhibiting mitochondrial damage and cell death.^21^ However, whether and how SIRT3 is involved in nephritis‐induced kidney injury remains unknown.

As an alpha‐2‐adrenoreceptor (α2‐AR) agonist, DEX has both sedative and analgesic functions.^22^ These agonizts are used as an alternative prophylactic approach for the prevention of sepsis.^23^, ^24^ α2‐ARs are distributed in peritubular tissues as well as in the proximal and distal tubules.^25^ Animal studies have revealed that DEX promotes the activation of α2‐ARs, thereby increasing glomerular filtration and renal blood flow. Additionally, clinical studies have shown that DEX can reduce perioperative (acute) renal injury.^26^, ^27^ As mentioned above, DEX has the ability to upregulate SIRT3 expression, leading to the inhibition of renal tubular epithelial cell death in animal models of I/R injury.^21^, ^28^ Hence, these studies suggest a possible link between DEX and SIRT3 in the mechanisms of renoprotection. Therefore, in the present study, we established both animal and cell‐based models of nephritis to examine whether DEX could alleviate renal injury in LN and to elucidate its role in the activation of SIRT3.

## 2 MATERIALS AND METHODS

### 2.1 Lentivirus generation

The short hairpin RNAs, shRNA‐Ctrl and shRNA‐SIRT3, were cloned in the lentiviral expression vector, pLVX‐Puro (Clontech), using phosphorylation and annealing processes and designated as pLVX‐shRNA‐Ctrl and pLVX‐shRNA‐SIRT3, respectively. These vectors were then separately used to infect HEK293T human embryonic kidney cells (Invitrogen) to generate lentiviral particles with pLVX‐shRNA (Ctrl‐shR) or pLVX‐shRNA‐SIRT3 plasmids along with pMD2.G and psPAX2.

### 2.2 Mouse model of nephritis

**Figure 1:**
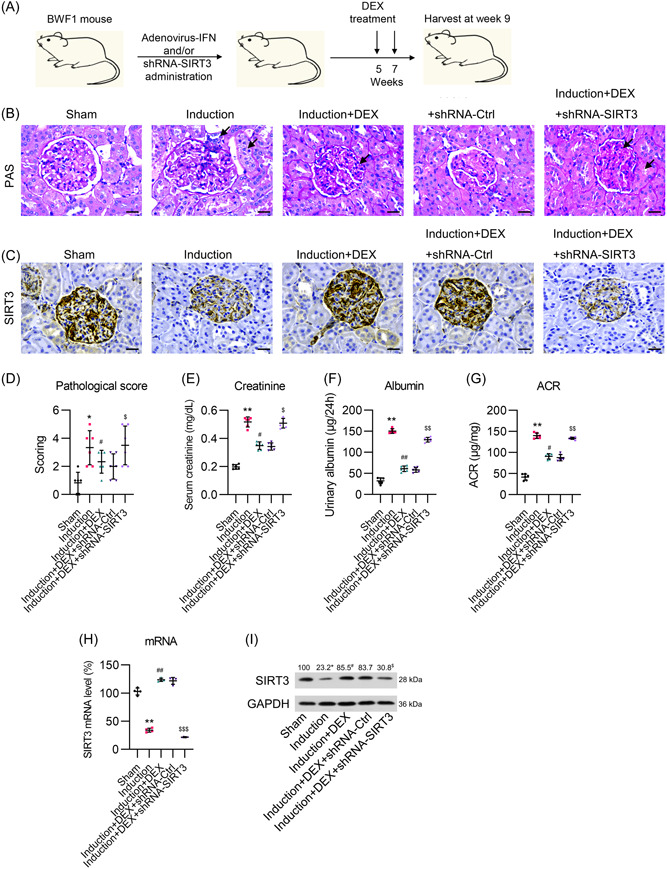
Effect of DEX on SIRT3 expression in the kidneys and its efficacy in treating IFNα‐accelerated lupus nephritis progression in mice. (A) Schematic diagram for timeline of animal treatment. (B) PAS‐stained kidney sections were analyzed for renal lesion scores in mice with IFNα‐accelerated lupus nephritis progression. Pathological changes were marked by black arrow. (C) Immunohistochemical, (D) Pathological scoring. (E) Serum creatinine, (F) Urine albumin, (G) Ratio of urine albumin to creatinine. (H) qPCR, and (I) western blot analyses of SIRT3 mRNA and protein expression in the kidneys of mice. DEX, dexmedetomidine; SIRT3, sirtuin 3. Scale bar, 50 μm. Number of replicates are 6. **p < .01; ^##^ *p* < .01; ^$$$^ *p* < .001.

A mouse model of nephritis was generated by infecting BWF1 mice with IFNα‐expressing adenoviruses. To obtain the BWF1 (NZB × NZW) population, C57BL/6 NZB and NZW mice (Vital River Laboratories, Beijing, China) were mated in cages in a specific‐pathogen‐free facility. Next, the BWF1 population (8–10 weeks old) resulting from the cross were administered a single intravenous injection of 10^9^ particles of IFNα‐expressing adenovirus 5 (hereinafter IFNα‐Ad5) (ViGene Biosciences) or the control adenovirus 5 for three consecutive days at 5 and 7 weeks of DEX treatment. The urine of mice injected with IFNα‐Ad5 was measured at 7 weeks after the last injection. For DEX treatment, mice were pretreated intraperitoneally with 10 mg/kg of the drug daily. The mice were euthanized and their kidneys were harvested at approximately 9 weeks after adenovirus injection in the treatment trial. Timeline for animal treatment was included in Figure 1A. All experiments were performed in accordance with the related laws and institutional guidelines and were approved by the Animal Studies Committee of Sir Run Run Shaw Hospital.

### 2.3 Cell cultures and modeling

The HEK293 cell line and human primary RMCs were purchased from the American Type Culture Collection and ScienCell Research Laboratories, respectively. The phenotypes of both cell lines were assessed each day in our laboratory. IFNα (1000 units/mL) from PBL InterferonSource (PBL Assay Science) was chosen for the induction of inflammation in the RMCs and HEK293 cells. For co‐treatment group, RMCs and HEK293 cells preincubated with 1 μM DEX were transfected with shRNA‐Ctrl or shRNA‐SIRT3 for 24 h and then treated with 1000 units/mL IFNα for another 24 h.

### 2.4 Cell transfection

The shRNA‐negative control (5′‐CAU GGC GAU UCA GUU AAU GCA U‐3′) and shRNA‐SIRT3 (5′‐AGG GUC GCC CCG UGA GUA ACG‐3′) oligonucleotides were purchased from Genscript Co., Ltd. Lipofectamine™ 3000 (Invitrogen) was used to transfect the shRNAs into the RMCs and HEK293 cells according to the manufacturer's instructions. Both cell lines were then used for subsequent experiments after 40 h of transfection.

### 2.5 Quantitative reverse transcription‐polymerase chain reaction assay (qPCR)

Total RNA was isolated from the kidney tissues and two cell lines using the RNeasy Mini Kit (Qiagen) according to the manufacturer's instructions. A NanoDrop™ ND‐1000 UV‐Vis spectrophotometer was then used to determine the RNA concentration. Then, 3 U/mg of RNase R (Epicenter) was used to digest a specific amount of RNA for 15 min at 37°C. The RNA (500 ng) was then reverse‐transcribed using the Prime Script™ RT Master Mix (Takara Bio Inc.) with oligo(dT) or arbitrary primers. qPCR was then carried out using the 2× PCR Master Mix (Thermo Fisher Scientific). The relative expression of the target genes was obtained using the delta‐delta Ct method, with the mRNA level of glyceraldehyde 3‐phosphate dehydrogenase (*GAPDH*) used as the internal control.

### 2.6 Enzyme‐linked immunosorbent assay

The cells subjected to different treatments were homogenized and kept on ice. The levels of the pro‐inflammatory cytokines interleukin‐1 beta (IL‐1β), interleukin‐6 (IL‐6), and tumor necrosis factor‐alpha (TNF‐α) were then evaluated using specific enzyme‐linked immunosorbent assay (ELISA) kits (Thermo Fisher Scientific) according to the manufacturer's instructions.

### 2.7 Measurement of reactive oxygen species

The fluorescence of 2ʹ,7ʹ‐dichlorofluorescein was used to measure reactive oxygen species (ROS) generation by the cells. In brief, the cell lines subjected to 1000 units/mL IFNα for 24 h and then reacted with 10 μM 2ʹ,7ʹ‐dichlorofluorescin diacetate for 30 min at 37℃ in the dark. The fluorescence intensity of the cells was then observed with a fluorescence microplate reader (M5, SpectraMax) at the excitation/emission wavelengths of 488/525 nm.

### 2.8 Measurement of antioxidative enzyme activities

The cell lines subjected to different treatments were homogenized and kept on ice. Next, the homogenate was centrifuged, and the supernatant was recovered and placed on ice. A standard BCA protein assay kit (Beyotime Institute of Biotechnology) was then used to determine the protein concentration in the supernatant. Thereafter, the activities of the antioxidative enzymes superoxide dismutase (SOD) and catalase were tested using the corresponding detection kits of Beyotime Institute of Biotechnology. The activity of each enzyme was expressed in units per milligram of tissue (U/mg).

### 2.9 MTT assay of cell viability

The viability of the cell lines subjected to different treatments was tested using the 3‐(4,5‐dimethylthiazol‐2‐yl)−2,5‐diphenyl‐2H‐tetrazolium bromide (MTT) assay. In brief, 20 µL of MTT (0.5 mg/mL) was added to the cells in a microplate. After the reaction period, the supernatant was discarded, and 150 μL of dimethyl sulfoxide was added to each well. The plate was then incubated for 10 min with shaking to dissolve the formazan dye. Finally, the absorbance of the contents in each well was measured at 540 nm using an Infinite M200 microplate reader (Tecan).

### 2.10 Colony formation assay

The effect of DEX treatment on cell viability was further investigated using the colony formation assay. In brief, at 2 days after transfection of the RMCs and HEK293 cells with the various shRNAs, the cells were resuspended in Dulbecco's modified Eagle's medium supplemented with 10% fetal bovine serum and spread over an 8 mL layer of 0.4% top agar. This was then transferred to 12‐well plates containing a 0.5‐mL layer of 0.5% bottom agar in each well. After 14 days, four regions were randomly selected from all plates for quantification of the colonies formed.

### 2.11 Flow cytometric assay

After culturing the cells subjected to various treatments for 48 h, the number of dead cells was evaluated using the Annexin V‐fluorescein isothiocyanate (FITC)/propidium iodide (PI) Apoptosis Assay Kit (Biyuntian Biotechnology Co. Ltd., Shanghai, China) according to the manufacturer's instructions. In brief, the cells were first suspended in Annexin V binding buffer (1×). Then, 1 μL of PI and 5 μL of Annexin V were added to 100 µL of the suspended cells, and the mixture was incubated for 15 min in the dark at ambient temperature. Next, 400 μL of 1× Annexin V binding buffer was added to terminate the reaction. The number of dead cells was counted using a FACSCalibur™ flow cytometer (BD Biosciences).

### 2.12 Western blot analysis

Total protein was extracted from the cells subjected to the different treatments using RIPA buffer (Beyotime Institute of Biotechnology) at 4℃. After resolution of the total proteins by electrophoresis, the protein bands were electrotransferred to polyvinylidene difluoride membranes for incubation with primary antibodies against the following target proteins: GAPDH (1:5000 dilution, ab8245, Abcam), caspase‐3 (1:1000, ab4051, Abcam), caspase‐9 (1:1000, ab25758, Abcam), cleaved caspase‐3 (1:1000, ab2302, Abcam), cleaved caspase‐9 (1:1000, ab2324, Abcam), Src homology region 2 domain‐containing phosphatase 1 (SHP‐1) (1:2000, ab227503, Abcam), signal transducer and activator of transcription 3 (STAT3) (1:2500, ab31370, Abcam), and phosphor STAT3 (1:500, ab7315, Abcam). Thereafter, the membranes were incubated with goat anti‐rat secondary antibody (1:1000, A0216, Beyotime Institute of Biotechnology) and horseradish peroxidase‐conjugated goat anti‐rabbit antibody (1:1000; A0208, Beyotime Institute of Biotechnology). The immune response zone was finally detected by development with a microporous chemiluminescence western blot kit (Millipore Sigma).

### 2.13 Statistical analysis

Data in the various figures are shown as the mean ± standard error of three independent experiments. SPSS software (version 17.0) was used for all statistical analyses. Data were evaluated by two‐tailed Student's *t*‐test or 1‐way ANOVA for comparison between two groups and among multiple groups. Differences with a *p* value of less than .05 were regarded as statistically significant.

## 3 RESULTS

### 3.1 Effect of DEX treatment on lupus nephritis development in the animal model

BWF1 mice were first infected with IFNα‐Ad5 and then intraperitoneally injected with DEX to study the potential effect of the drug on alleviating LN. Histological examination of the extracted mouse kidney tissue was achieved through periodic acid–Schiff staining. The results showed that the IFNα‐Ad5‐infected mice had developed severe glomerulonephritis. Importantly, DEX‐administered mice developed healthy glomeruli without significant glomerular enlargement or mesangial cell proliferation (Figure 1B). The immunohistochemical (IHC) staining, qPCR, and western blot results showed that DEX administration led to the upregulation of SIRT3 expression in the kidney tissue of the mice with LN (Figure 1B–D). To investigate the effect of SIRT3 expression on the renoprotective effect of DEX on mice with LN, the animals were pre‐injected with shRNA‐SIRT3 to knockdown the expression of *SIRT3*. As expected, shRNA‐SIRT3 administration significantly downregulated SIRT3 levels in the kidney tissue, as assessed by IHC, qPCR, and western blot assays (Figure 1C–I). Moreover, *SIRT3* knockdown accelerated damage to the glomeruli in the kidney tissue, as indicated by images and scoring (Figure 1B,D). The examination on serum creatinine, urine albumin, and ratio of urine albumin to creatinine indicated that *SIRT3* knockdown increased renal damage in the kidney tissue of animals (Figure 1E–G). These data suggested that DEX alleviated kidney injury caused by LN in the mouse model, probably by promoting SIRT3 expression.

### 3.2 SIRT3 expression was regulated in DEX‐treated HEK293 cells and renal mesangial cells

**Figure 2:**
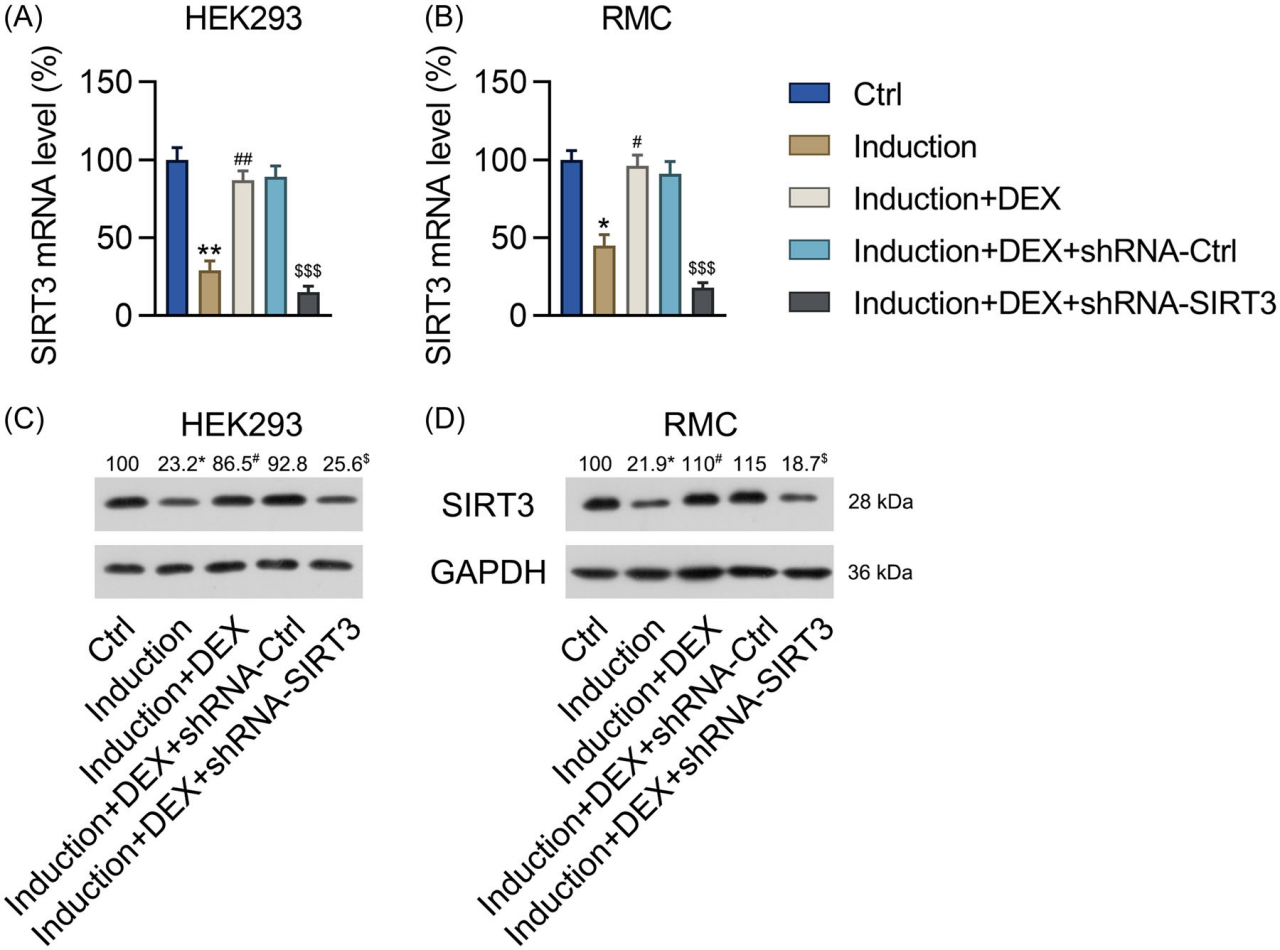
Effect of DEX on SIRT3 expression in RMCs and HEK293 cells with IFNα‐induced lupus nephritis. RMCs and HEK293 cells preincubated with 1 μM DEX were transfected with shRNA‐Ctrl or shRNA‐SIRT3 for 24 h and then treated with 1000 units/mL IFNα for another 24 h. (A, B) The SIRT3 mRNA expression levels in the RMCs and HEK293 cells were detected with the qPCR assay. (C, D) The SIRT3 protein expression levels in the RMCs and HEK293 cells were detected with the western blot assay. DEX, dexmedetomidine; SIRT3, sirtuin 3; RMCs, renal mesangial cells; HEK293, human embryonic kidney cell line. Number of replicates are 3. *p < .05, **p < .01; ^#^ *p* < .05, ^##^ *p* < .01; ^$$$^ *p* < .001.

Mesangial cells and HEK293 cells are two cell lines to investigate the LN progression, and to confirm the role and finding of DEX in suppressing LN progression and the possible mechanism involved. HEK293 cells and RMCs were preincubated with the drug, following which the LN phenotype was induced by treating them with IFNα. Notably, IFNα treatment reduced the expression of SIRT3 in cells that had not been pretreated with DEX, whereas SIRT3 expression at the protein and mRNA levels was increased in the cells that received DEX pretreatment (Figure 2A–D). To determine the role of SIRT3 in protection against IFNα‐induced LN, RMCs and HEK293 cells were transfected with shRNAs to silence the expression of *SIRT3*, and the effects on various aspects of LN were investigated (as described in the following sections below). The qPCR and western blots results confirmed that the expression of *SIRT3* had been significantly reduced in both the RMCs and HEK293 cells following their transfection with shRNA‐SIRT3 (Figure 2A–D). In SIRT3 knockout samples, the increased lysine acetylation level was found indicating the SIRT3 activity change (Figure S1).

### 3.3 *SIRT3* silencing abolished effect of DEX in IFNα‐treated RMCs and HEK293 cells

**Figure 3:**
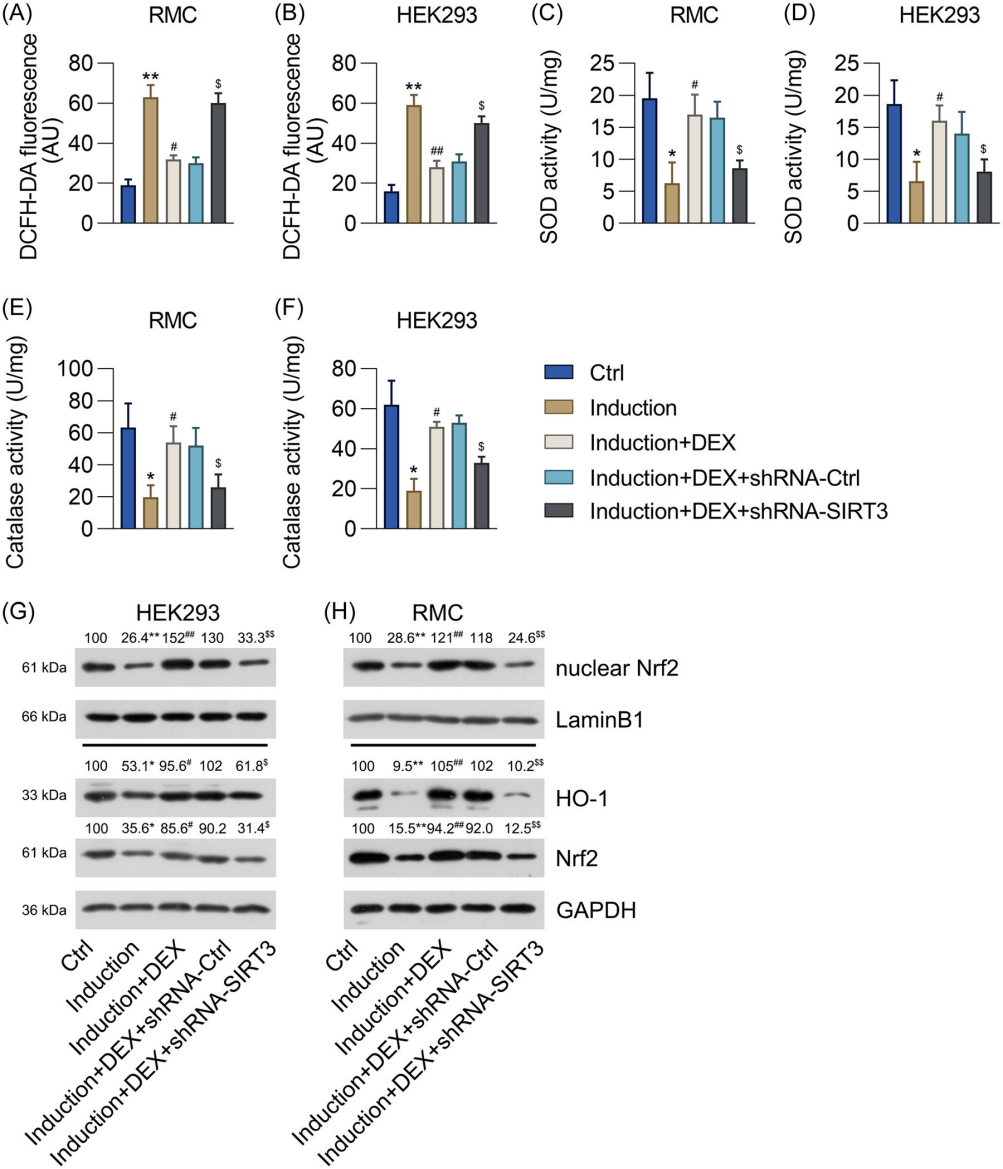
Effect of *SIRT3* silencing on oxidative stress in DEX‐pretreated RMCs and HEK293 cells with IFNα‐induced lupus nephritis. RMCs and HEK293 cells preincubated with 1 μM DEX were transfected with shRNA‐Ctrl or shRNA‐SIRT3 for 24 h and then treated with 1000 units/mL IFNα for another 24 h. (A) DCFH‐DA staining was performed to evaluate the production of ROS. (B, C) The activities of the antioxidative enzymes SOD and catalase were determined using the corresponding assay kits. (D) The protein levels of Nrf2, nuclear Nrf2, and HO‐1 in the cells were detected with the western blot assay. DEX, dexmedetomidine; SIRT3, sirtuin 3; RMCs, renal mesangial cells; HEK293, human embryonic kidney cell line; ROS, reactive oxygen species; SOD, superoxide dismutase; HO‐1, heme oxygenase‐1, Nrf2, nuclear factor‐erythroid factor 2‐related factor 2. Number of replicates are 3. *p < .05, **p < .01; ^#^ *p* < .05, ^##^ *p* < .01; ^$^ *p* < .05.

In patients with SLE, ROS interact with lipids, proteins, and nucleic acids as well as bicarbonates to promote immune regulation and trigger autoimmunity, leading to chronic and acute tissue damage. Oxidative stress in nephritis can result in robust inflammation in renal cells and their eventual death.^29^ We first determined whether IFNα could induce oxidative stress in RMCs and HEK293 cells, and how DEX and SIRT3 are involved in the process, by determining the levels of ROS production, SOD and catalase activities, and nuclear factor‐erythroid factor 2‐related factor 2 (Nrf2)/heme oxygenase‐1 (HO‐1) signal transduction in the *SIRT3*‐silenced cells. It was found that IFNα increased ROS generation and reduced the SOD and catalase activities in both RMCs and HEK293 cells transfected with shRNA‐Ctrl, whereas DEX treatment alleviated the IFNα‐induced oxidative stress in those cells (Figure 3A–F). Moreover, *SIRT3* silencing counteracted this effect of DEX, with the cells showing high levels of ROS generation and reduced SOD and catalase activities (Figure 3A–F). We also observed that the Nrf2, nuclear Nrf2, and HO‐1 protein levels were reduced by IFNα in the control cells, whereas DEX treatment reactivated the Nrf2/HO‐1 signaling pathway (Figure 3G,H). However, *SIRT3* silencing canceled the effects of DEX on these changed protein levels (Figure 3G,H). These data suggest that DEX repressed oxidative stress in the IFNα‐treated RMCs and HEK293 cells by regulating SIRT3 expression.

### 3.4 *SIRT3* participated in the DEX alleviation of inflammation in IFNα‐treated RMCs and HEK293 cells

**Figure 4:**
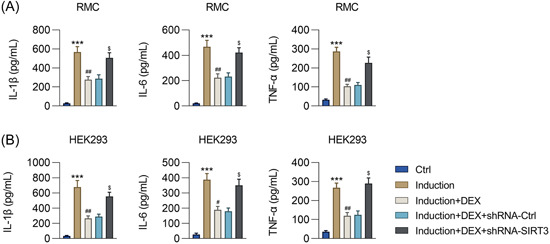
Effect of *SIRT3* silencing on inflammation in DEX‐pretreated RMCs and HEK293 cells with IFNα‐induced lupus nephritis. RMCs and HEK293 cells preincubated with 1 μM DEX were transfected with shRNA‐Ctrl or shRNA‐SIRT3 for 24 h and then treated with 1,000 units/mL IFNα for another 24 h. ELISA was performed to detect the release of IL‐1β, IL‐6, and TNF‐α into the media by the (A) RMCs and (B) HEK293 cells. DEX, dexmedetomidine; SIRT3, sirtuin 3; RMCs, renal mesangial cells; HEK293, human embryonic kidney cell line; IL‐1β, interleukin‐1 beta; IL‐6, interleukin‐6; TNF‐α, tumor necrosis factor‐alpha. Number of replicates are 3. ***p < .001; ^#^ *p* < .05, ^##^ *p* < .01; ^$^ *p* < .05.

Next, we tested the effects of DEX treatment and *SIRT3* silencing on the inflammatory response in the two cell lines with the IFNα‐induced LN phenotype. ELISA was used to examine the expression and release of pro‐inflammatory cytokines (IL‐1β, IL‐6, and TNF‐α) by the cells. IFNα upregulated the levels of all three cytokines in both types of cells, but this effect was alleviated with DEX administration. Transfection with shRNA‐SIRT3 contributed to a considerable increase in the levels of these three cytokines compared with the levels in the shRNA‐Ctrl group (Figure 4A,B), indicating that *SIRT3* silencing counteracted the inhibitory role of DEX against IFNα‐induced inflammation in HEK293 cells and RMCs.

### 3.5 SIRT3 was involved in DEX‐reduced cell death and DEX‐promoted cell survival in IFNα‐treated HEK293 cells and renal mesangial cells

**Figure 5:**
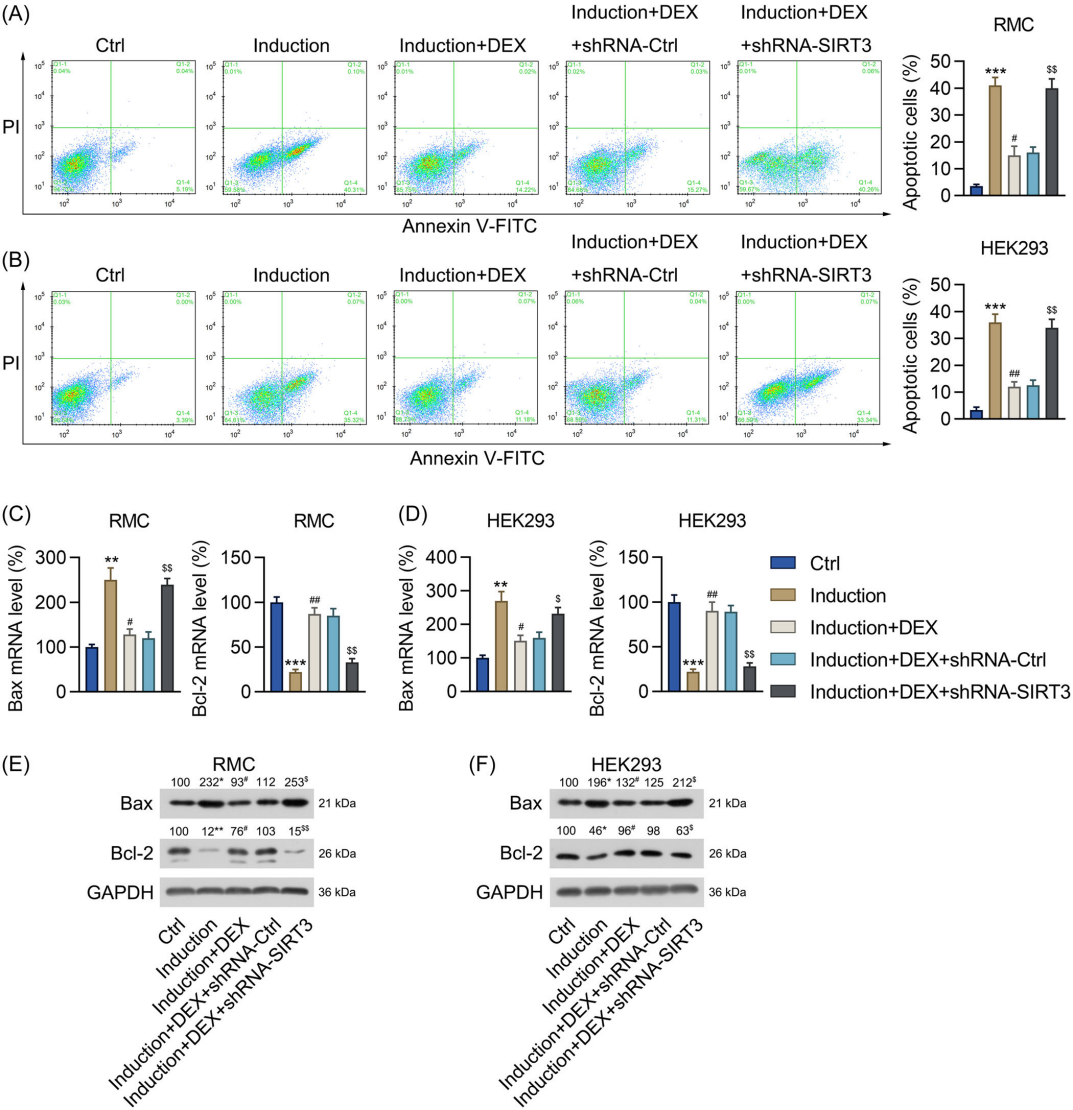
Effect of *SIRT3* silencing on apoptosis in DEX‐pretreated RMCs and HEK293 cells with IFNα‐induced lupus nephritis. RMCs and HEK293 cells preincubated with 1 μM DEX were transfected with shRNA‐Ctrl or shRNA‐SIRT3 for 24 h and then treated with 1,000 units/mL IFNα for another 24 h. (A, B) Annexin V‐FITC/PI flow cytometric assay of the proportion of apoptotic RMCs and HEK293 cells. (C, D) The mRNA expression levels of Bax and Bcl‐2 in the RMCs and HEK293 cells were assessed using the qPCR assay. (E, F) The protein levels of Bax and Bcl‐2 in the RMCs and HEK293 cells were assessed using the western blot assay. DEX, dexmedetomidine; SIRT3, sirtuin 3; RMCs, renal mesangial cells; HEK293, human embryonic kidney cell line; Bax, Bcl‐2‐like protein 4; Bcl‐2, B‐cell lymphoma 2. Number of replicates are 3. **p < .01, ***p < .001; ^#^ *p* < .05, ^##^ *p* < .01; ^$^ *p* < .05, ^$$^ *p* < .01.

Considering that DEX reversed IFNα‐induced oxidative stress and inflammation (known to be two major causes of apoptosis)^30^ in RMCs and HEK293 cells, we hypothesized that SIRT3 may also be involved in reducing the apoptosis induced by IFNα in these two cell lines. According to the Annexin V‐FITC/PI flow cytometry results, IFNα significantly induced cell death, whereas DEX treatment significantly reduced the percentage of apoptotic cells in the shRNA‐Ctrl‐transfected cell lines. Furthermore, *SIRT3* silencing reversed the inhibitory effect of DEX on apoptosis (Figure 5A,B). Additionally, the IFNα‐treated control cells showed reduced Bcl‐2 and increased Bax expression levels, whereas DEX treatment abolished these IFNα‐induced effects. Moreover, *SIRT3* knockdown resulted in a low level of Bcl‐2 expression and a high level of Bax expression in the DEX‐ and IFNα‐treated RMCs and HEK293 cells (Figure 5C–F).

**Figure 6:**
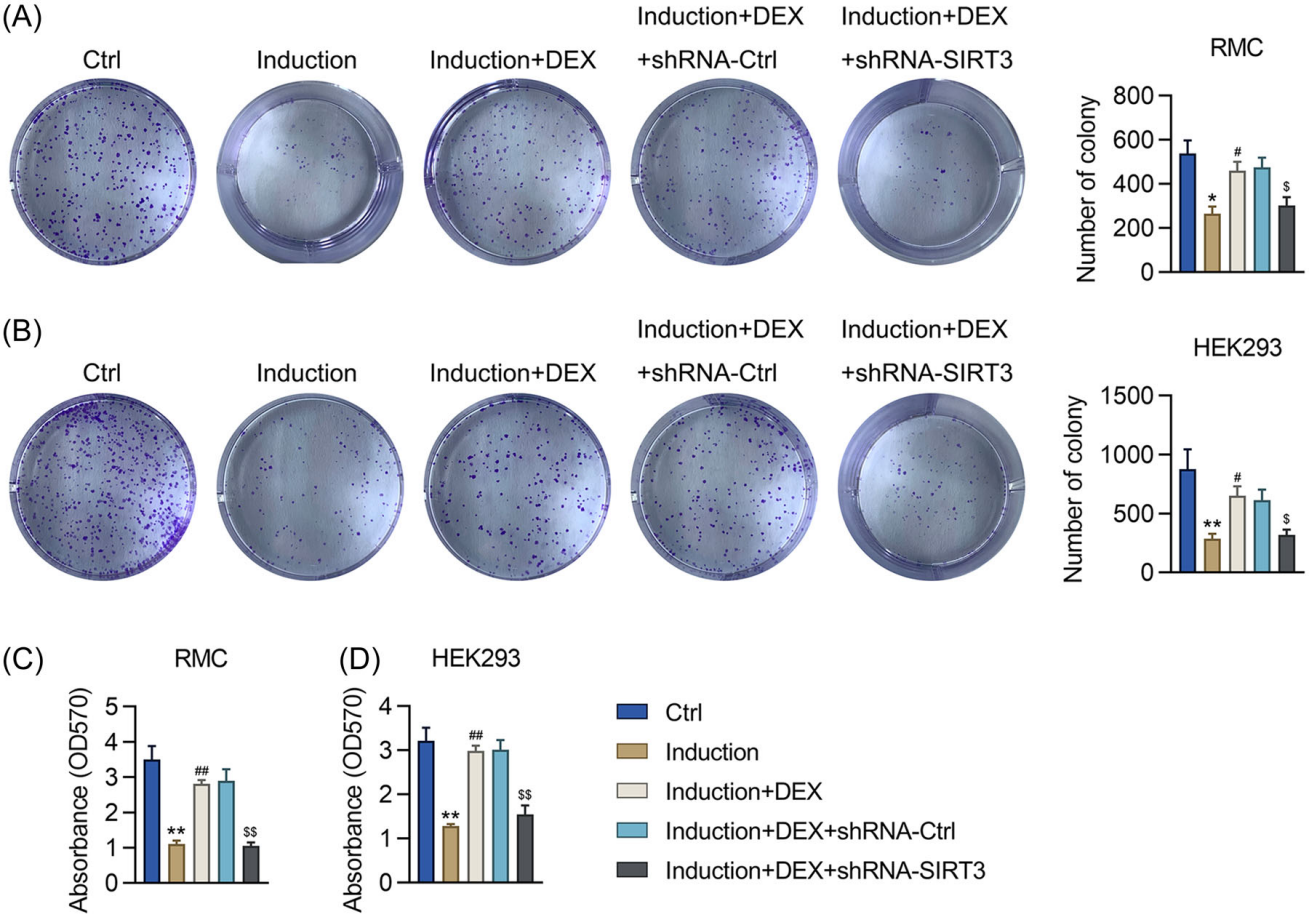
Effect of *SIRT3* silencing on the viability of DEX‐pretreated RMCs and HEK293 cells with IFNα‐induced lupus nephritis. RMCs and HEK293 cells preincubated with 1 μM DEX were transfected with shRNA‐Ctrl or shRNA‐SIRT3 for 24 h and then treated with 1,000 units/mL IFNα for another 24 h. (A, B) The proliferation rate of the cells was measured at 96 h post‐IFNα treatment using the colony formation assay. (C, D) The cell survival rate was determined using the MTT assay. Results are expressed as the mean ± SEM. *p < .05, **p < .01 versus indicated group. DEX, dexmedetomidine; SIRT3, sirtuin 3; RMCs, renal mesangial cells; HEK293, human embryonic kidney cell line. Number of replicates are 3. *p < .05, **p < .01; ^#^ *p* < .05, ^##^ *p* < .01; ^$^ *p* < .05, ^$$^ *p* < .01.

Finally, the effect of DEX on the viability of IFNα‐treated HEK293 cells and RMCs was analyzed using the colony formation assay and MTT assay. IFNα significantly decreased the number of colonies formed and the survival rate of both shRNA‐Ctrl‐transfected HEK293 cells and RMCs, whereas the pretreatment of these cells with DEX alleviated these effects. Moreover, *SIRT3* knockdown reversed the influence of DEX on the viability of the IFNα‐treated cells (Figure 6A–D). These results indicate that SIRT3 expression is required for DEX to exert its renoprotective effects in IFNα‐treated HEK293 cells and RMCs.

## 4 DISCUSSION

On the basis of previous in vitro and in vivo studies, we know that SIRT3 is somehow involved in the protective effects of the receptor agonist DEX against the damage from nephritis caused by I/R injury,^28^ prompting us to investigate the nature of this involvement more clearly. To this end, we established both an in vivo animal model and an in vitro cell‐based model of LN in this study. We observed that the cell damage induced by IFNα (oxidative stress, inflammation, and apoptosis) could be suppressed after intraperitoneal DEX administration in the mouse model and with DEX preincubation in the cell model. DEX treatment increased the SIRT3 expression levels both in vivo and in vivo. By contrast, *SIRT3* gene knockdown partially abolished the renoprotective effects of DEX on the pathogenic changes in mice with LN. Moreover, the silencing of *SIRT3* in the DEX‐pretreated and IFNα‐induced cells resulted in increases in the generation of ROS and pro‐inflammatory cytokines and in the number of apoptotic cells as well as decreases in the growth and survival rate of the cells, suggesting that the influence of DEX on IFNα‐treated cells might, at least in part, be SIRT3 dependent.

IFNα is a key cytokine in the pathogenesis of LN. Moreover, elevated levels of pro‐inflammatory cytokines (e.g., IL‐1β, IL‐6, and TNF‐α) are related to the disease activity and severity of SLE.^31^ The inoculation of lupus‐susceptible or RMC‐bearing mice with IFNα (exogenous) increased the severity and rapid progression of LN, suggesting that IFNα has a pathogenic role in the development of this condition,^32^, ^33^, ^34^ although the exact mechanisms involved remains unknown. In this study, HEK293 cells and RMCs were treated with IFNα to build a cell model of LN, and mice were also injected with the cytokine to establish an animal model of the disease. The notable pathological changes in the renal tissue of the mice and the impaired viability of the cell lines suggested the successful establishment of both animal and cell models.

Because DEX has good analgesic, sedative, sympathetic neurolysis, and hemodynamic effects, it is widely used in clinical practice, especially in the perioperative period, and it also acts as an anti‐inflammatory, antioxidative, and antiapoptotic agent for vital organs (e.g., heart, brain, lungs, and kidneys).^25^, ^26^, ^35^, ^36^ DEX has been shown to protect renal cells against I/R damage both in vivo and in vitro. In animal models, DEX could inhibit the death of renal tubular epithelial cells, thereby reducing renal injury.^21^ Other studies have shown DEX to inhibit the inflammatory response and partially alleviate the renal and myocardial damage caused by renal I/R injury in a dose‐dependent manner^37^; to reduce vacuolation and mitochondrial swelling of the dorsal root ganglion caused by neuropathic pain^38^; and to alleviate oxidative stress‐induced alveolar epithelial cell death by restraining ROS generation.^36^ Li et al. found that DEX preconditioning protects against myocardial ischemia/reperfusion injury by exerting antioxidant stress through activation of the Keap1/Nrf2 signal transduction pathway, while inhibition of the Keap1‐Nrf2/ARE signal transduction pathway reverses the protective effect of DEX preconditioning on myocardial ischemia/reperfusion injury.^39^ Liu et al. indicated that DEX relieves neuropathic pain in rats with chronic constriction injury via the Keap1/Nrf2 Pathway.^40^ In our study and that of others, DEX was found to have a beneficial effect in protecting against kidney injuries in patients undergoing major operations, such as cardiovascular surgery.^41^, ^42^ However, the role of DEX in LN remains unknown. Our findings suggest that DEX downregulates cellular ROS generation, upregulates SOD and catalase activities, activates Nrf2/HO‐1 signal transduction, and reduces inflammation and apoptosis in nephritis. Overall, DEX can inhibit oxidative stress, inflammation, and apoptosis to promote cell viability.

Previous studies have confirmed the role of SIRT3 as a protectant against acute kidney tissue injury.^20^ In one study that used cisplatin to induce acute kidney injury in mice, the mitochondrial damage and increased oxidative stress that ensued were found to be related to reduced renal SIRT3 expression (~80%).^20^ SIRT3 is a mitochondrial protein deacetylase. In SIRT3‐deficient mice, mitochondrial proteins are highly acetylated.^43^, ^44^, ^45^ According to previous studies, during myocardial I/R injury as well as under renal oxidative stress, SIRT3 exerts a beneficial effect by regulating the opening of cyclophilin d‐dependent mitochondrial permeability transition pores.^46^ The targeting of SIRT3 effectively reduced both mitochondrial fragmentation and the degree of damage to the kidneys and accelerated their functional recovery. These results suggest that maintenance of the mitochondrial structure and function can promote stable recovery from renal injury.^20^, ^47^, ^48^ In a murine model of *SIRT3* gene knockout, fibrosis developed in the lungs, liver, and kidneys of mice at 15 months of age.^49^ Several previous studies demonstrated a direct regulatory loop between SIRT3 and Nrf2 in various diseases,^50^, ^51^, ^52^, ^53^ indicating that SIRT3‐Nrf2 loops also exerted its function in LN development. Our results from the RMC and HEK293 cell models concurred with previous findings that SIRT3 upregulation following DEX pretreatment is positively correlated with alleviated inflammation and oxidative stress and reduced cell death. Moreover, *SIRT3* knockdown in mice partially abolished the renoprotective effects of DEX against LN.

## 5 CONCLUSION

In conclusion, DEX treatment reduced renal injury caused by nephritis in a mouse model of LN and alleviated damage (inflammation, oxidative stress, and cell death) in IFNα‐treated RMCs and HEK293 cells. During LN model development, DEX significantly promoted SIRT3 expression in the kidneys and cell lines. Further findings showed that the renoprotective effects of DEX on these in vivo and in vitro models were partially SIRT3‐dependent. Therefore, we concluded that DEX prevents nephritis by upregulating SIRT3 expression. The results of this study offer supporting information on the potential mechanism of DEX in its protection of the kidneys in LN as well as the involvement of SIRT3 in this process.

## AUTHOR CONTRIBUTIONS

**Kai Lu**: Conceptualization; writing—original draft; writing—review and editing. **Xinlong Li**: Conceptualization; data curation; formal analysis; investigation; validation. **Jie Wu**: Data curation; formal analysis; investigation; validation.

## CONFLICT OF INTEREST STATEMENT

The authors declare no conflict of interest.

## Supporting information


**Supplementary figure 1**. Effect of SIRT3 silencing on total protein lysine acetylation level in whole cell lysate.

## Data Availability

The data that support the findings of this study are available from the corresponding author upon reasonable request.
