# Supplementary figures and images for: Sirtuin 3 is required for the dexmedetomidine‐mediated alleviation of inflammation and oxidative stress in nephritis
Source: Immun Inflamm Dis. 2024 Jan 10;12(1):e1135. doi: 10.1002/iid3.1135 (PMC10777884; doi:10.1002/iid3.1135)

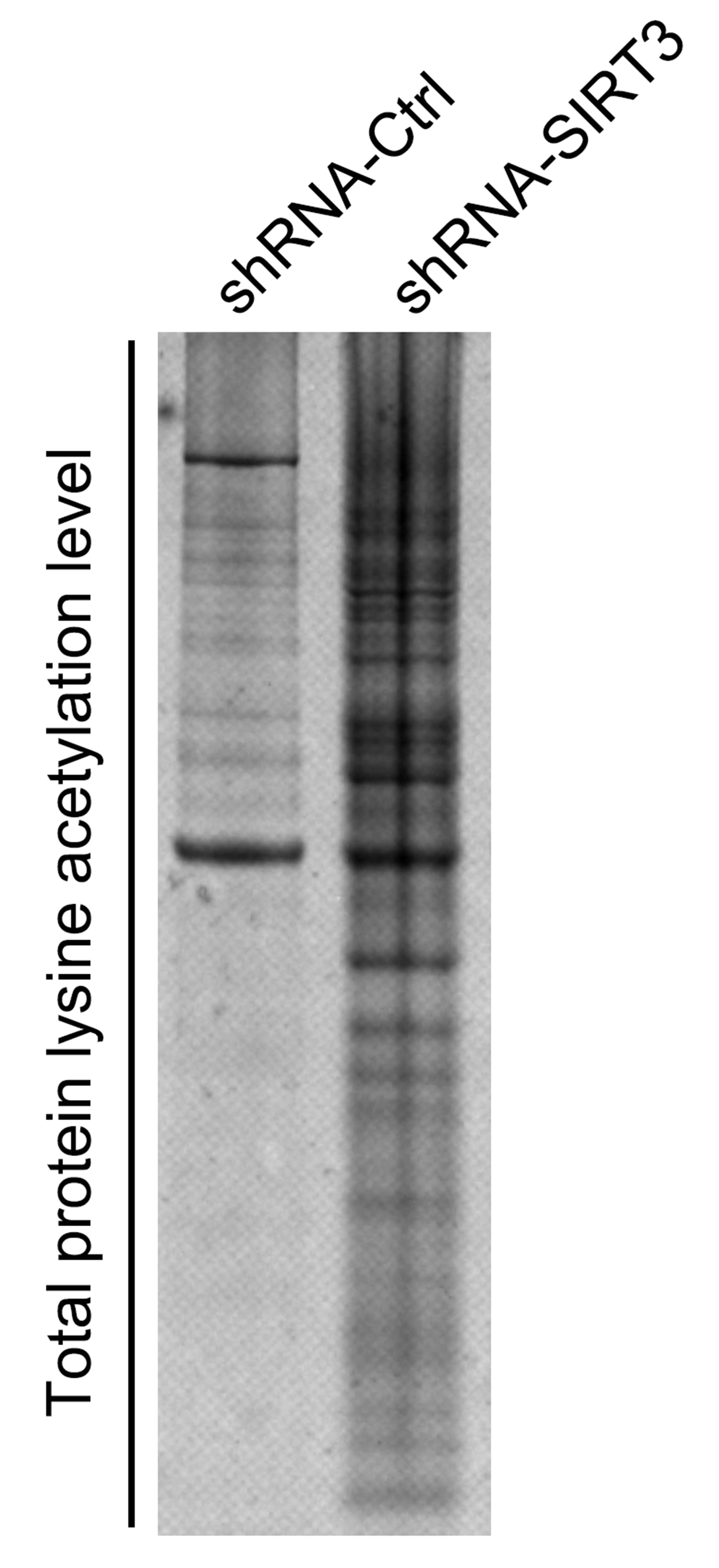

Supplement: Supplementary file 1 — Supplementary figure 1. Effect of SIRT3 silencing on total protein lysine acetylation level in whole cell lysate. [file IID3-12-e1135-s001.tif]
